# Factors That Influence the Intention of Smallholder Rice Farmers to Adopt Cleaner Production Practices: An Empirical Study of Precision Agriculture Adoption

**DOI:** 10.1177/0193841X231200775

**Published:** 2023-09-07

**Authors:** Long Le Hoang Nguyen, Duong Thuy Khuu, Alrence Halibas, Trung Quang Nguyen

**Affiliations:** 1 113073 RMIT University Vietnam, Ho Chi Minh, Vietnam; 2 WWF-Viet Nam, Ha Noi, Vietnam

**Keywords:** sustainable agriculture, precision agriculture, sustainable development goals, cooperatives, lead firms, climate change, developing countries, technology adoption, unified theory of acceptance and use of technology

## Abstract

Sustainable agriculture is crucial for achieving SDG2 and building a resilient climate-food system. This study provides a nuanced understanding of factors that influence the adoption of precision agriculture technology by Vietnamese smallholder rice farmers as a sustainable agricultural solution. The study’s objectives are: (1) to provide a nuanced understanding of factors that influence adoption of precision agriculture technology by Vietnamese smallholder rice farmers; and (2) to identify factors associated with agricultural practice in Vietnam that may impact the adoption of precision agriculture technology. The study conducted 35 semi-structured interviews with smallholder rice farmers and agriculture experts. Data were analyzed using iterative thematic analysis. The Unified Theory of Acceptance and Use of Technology (UTAUT) model was used for empirical analysis. The UTAUT constructs of Performance expectancy, Effort expectancy, Government support, and Social influence were found to be determinants of adoption. Trialability and Observability impact Effort expectancy and Performance expectancy. We also discovered that the performance of agricultural cooperatives and support of lead firms play a crucial role in facilitating agricultural technology adoption by Vietnamese smallholder rice farmers. The results confirm the applicability of UTAUT in defining smallholders’ behavioural intention and predicting agricultural technology adoption. The study also provides constructive feedback on the UTAUT model by highlighting the effect of agricultural cooperatives’ performance as innovation intermediaries and of the support of lead firms.

## Introduction

Feeding a growing human population and meeting the United Nations’ Sustainable Development Goals require the structural transformation of agricultural production towards higher productivity and sustainability. Precision agriculture (PA) technology provides a promising tool to achieve this transformation. Its application also aligns with the regional sustainable development goal, which is associated with driving technological innovations for controlling environmental impacts (Liu et al., 2022). To date, PA adoption studies have provided avenues for governments, farming associations, and technology vendors to improve precision technology use in agricultural sectors (Aubert et al., 2012; Duncan et al., 2021; Li et al., 2020). However, a lack of farmer acceptance and participation in that adoption is often a key barrier to the PA practice success. Therefore, a good understanding of farmers’ behavioural intention to adopt PA technology is essential for policymaking, especially in developing countries, where socio-economic and engineering conditions present real challenges to technology adoption (Mondal & Basu, 2009; Panwar et al., 2021).

Vietnam is a key player in global agricultural production. In 2021, it was ranked the world’s second-largest rice exporter, with 6.2 million tons of rice exported in 2020 (14% of global rice export volume) (USDA, 2021). However, Vietnamese rice farmers face problems of low productivity, shrinking cultivation areas, and inefficient input usage. Although rice accounts for 94% of land under intensive cultivation (BritCham Vietnam, 2021), it contributes only 25% of national agricultural production (OECD, 2020b). This partly explains why around 200,000 ha of inefficient rice-growing land have been converted to other crops offering higher economic benefits (PSAV, 2018). Decreasing rice cultivation area will continue in Vietnam and other countries in the Asia-Pacific region (Joshi, 2021). Therefore, to remain a top rice exporter, Vietnam must improve rice production efficiency to respond to local competition from other crops for cultivated land. Overuse of fertilizers in Vietnamese rice farming reveals a practical need for initiatives to enhance nutrient input efficiency and reduce environmental costs. Fertilizer is the largest single-cost item for crops, especially rice, in Vietnam (Liu & Wu, 2021). The country annually consumes about 7 million tons of fertilizer in rice farming (World Bank Group, 2016). However, around 30–40% of this is wasted (Truong et al., 2021). This research is thus timely, as it provides insight into factors influencing farmer behavioural intention to adopt precision agriculture technology, which is critical for improving rice-farming productivity and efficiency. This is particularly important given the urgent need to retain farmers in the rice-farming sector and growing government interest (OECD, 2020b) in promoting agricultural sustainability.

Several studies have addressed factors affecting adoption of general ICT applications for farming in Vietnam (Lampach et al., 2021; Tran et al., 2019) and other developing countries (Burkitbayeva et al., 2020; Kante et al., 2019; Tolentino & Hernandez, 2020). However, these findings do not fully explain the complex, unique nature of PA (Nguyen et al., 2022): while other general ICTs require only a single system or device installation, PA technology often requires a systematic combination of diagnostic tools (GPS, GIS, and remote sensing) and applicative tools (variable rate application, unmanned vehicles) to ensure maximum benefits from synergizing crop monitoring and precise control of nutrient inputs (Chawade et al., 2019; Zhao et al., 2020). PA technology adoption will shift Vietnamese farming practices towards focussing on leveraging the power of farming data analysis for precise chemical and nutrient treatment (Monteleone et al., 2019) to achieve productivity and environmental sustainability.

This study, through an empirical lens, seeks to identify and understand factors that affect Vietnamese smallholder rice farmers’ intention to adopt PA technology, proposing the following research questions (RQs):

RQ 1 How are the factors influencing the behavioural intention of farmers to adopt precision agriculture are being perceived?

RQ 2 How are these factors related to the current agricultural practices in Vietnam?

We use the iterative thematic analysis approach, building on the theoretical underpinning of the well-known Unified Theory of Acceptance and Use of Technology (UTAUT) model by Venkatesh et al. (2003) as guiding themes to investigate different views of stakeholders (farmers, the state, technology developers, and activists) involved in PA adoption. To ensure population representation of our sample, we have first adopted a stratified purposeful sampling strategy (Suri, 2011) by clearly considering multiple participant groups and specifying selection criteria for each. Second, selection of farmers and experts was based on geographical representation (North, Central, and South Vietnam) and farm sizes to ensure participant profile diversity (Saunders et al., 2016). Data saturation was addressed by ensuring that new data and insights no longer emerged from the sample (Hennink & Kaiser, 2022) (see the Methodology section, below).

This study contributes theoretically to the PA adoption literature with an emphasis on agricultural cooperatives’ role as innovation intermediaries, whereas most studies on cooperatives have only discussed whether or not farmers are members (Barnes et al., 2019; Ruzzante & Bilton, 2018; Takácsné et al., 2018). We also highlight the role that lead firms play in the adoption of PA in Vietnam, which has been largely ignored in the literature. It is argued that the key difference between findings on PA adoption in Vietnam and in other countries is that Vietnam’s agricultural land is more fragmented than other regional countries such as Cambodia, Myanmar, and the Philippines (Nguyen et al., 2020). Average rice farm size in Vietnam is approximately 1.2 ha (Ninh, 2021). This high level of land fragmentation poses a unique challenge for farmers wishing to adopt modern mechanized equipment, as previous studies report that land fragmentation is a barrier to technology adoption (Tran & Van Vu, 2019). This suggests potential findings about the importance of linkage and external facilitating conditions in fostering PA adoption.

Practically speaking, this paper contributes to PA technology development and diffusion as an emerging innovation in developing countries’ agri-food system and agricultural practice. Given PA incentives of profitability and environmental sustainability, opportunities arise for entrepreneurs to add value to developing-country farming practice, where PA technologies are still relatively a new concept. Efforts from governments, technology developers, and farmers to facilitate a higher PA adoption rate align with the farming innovation and technology advancement agenda for rural development in many countries.

## Literature Review

### The Status of Technology Application for Vietnamese Cleaner Agricultural Practices

Recognizing that chemical input overuse in Vietnam’s agricultural sector is a key weakness in farming practice, the OECD (2020b) suggests further efforts be made to control the input level in agricultural production to improve sector competitiveness and sustainability. Vietnam now faces increasing negative environmental impact through crop growth intensification (Kiet et al., 2021). Vietnam’s agriculture has featured heavy and inefficient use of fertilizer and pesticides. More than 10 million tons of fertilizer is used annually, two-thirds of that for rice (World Bank Group, 2016). Excess fertilizer and poor water management lead to considerable fertilizer run-off into streams or groundwater, causing severe water pollution (Schreinemachers et al., 2020).

Vietnam is also a heavy pesticide user, despite government efforts to encourage integrated pest management solutions (OECD, 2020b). Pressure to maximize crop growth has increased use since the 2000s (World Bank Group, 2016). This has led to more frequent sampling and testing in strict markets. Pesticide waste negatively affects the environment and brings severe health-related risks to Vietnamese farmers (OECD, 2020b), hence the need for better practices and technology applications.

Technology application is considered the most effective approach to agriculture sector sustainability due to its potential to deal with national concerns for improving productivity but maintaining the ability to preserve the environment (Luong et al., 2019) and enabling supply chain sustainability (Akbari & Hopkins, 2022; Ghobakhloo, 2020). However, the current technology application status in agriculture does not paint a favourable picture. About 66% of farms rely on manual harvesting; only 10.8% use machinery (Cirera et al., 2021). The World Bank reports that advanced irrigation systems are mostly used by larger-size-agricultural firms of larger size and are unpopular amongst most Vietnamese farming households (Cirera et al., 2021; Tolentino & Hernandez, 2020). Vietnam’s high-tech agriculture lags behind other countries, despite a long agricultural history. With many traditional smallholder farmers, high-tech agriculture remains a relatively unknown concept (Tran & Pham, 2020).

The government has consequently pledged numerous incentives to private firms, cooperatives, and farmers to foster investment in high-tech agricultural solutions (OECD, 2020b). In 2018, the master plan for developing high-tech agricultural zones by 2020 through to 2030 was approved: it aimed to develop large-scale, modern agricultural production through leveraging mechanization and advanced technology in domestic agriculture (Kawarazuka & Prain, 2019). Currently, such products contribute only 25% of total agricultural product value, leaving room for future growth (Shira, 2021).

### Nuanced PA Conceptualization

PA was introduced as a new farm management concept in the mid-1980s, but there is no universal conceptualization (Robert, 2002). PA can be perceived as the use of technologies and management strategies to manage all aspects of agricultural production to achieve crop performance and environmental sustainability (Pierpaolia et al., 2013). To provide a more comprehensive understanding of PA, many researchers further clarified and extended the PA concept regarding its relevant technologies, practices, and benefits. Bongiovanni and DeBoer (2004) conceptualized PA with the use IT applications to monitor soil and crop conditions electronically, to target treatment in detail. This way of conceptualization has been agreed upon and adopted by later studies in all aspects of PA concerning relevant technologies (Pierpaolia et al., 2013), for economic outcomes (Colaço & Bramley, 2018), environmental effects (Kelc et al., 2019), and adoption drivers (Li et al., 2020). The term ‘PA’ has become interchangeable with site-specific crop management (Rogers et al., 2016) and precision farming (Godwin et al., 2003), which represent the same notion of farming management based on monitoring, measuring, and responding to ‘intra-field variability’ in agricultural production (Lindblom et al., 2017, p. 309).

In Vietnam, PA research has been limited to technology’s descriptive analysis and feasibility assessment; in practice, as noted, the status of applications in agriculture does not look positive. In 2017, the Prime Minister approved a credit package of USD 4.4 billion for high-tech agriculture application loans (BritCham Vietnam, 2021), but this still challenges small farming households’ credit applications due to their micro-scale and lack of collateral (Vu & Ho, 2022). Thus, from 2018 to 2020, the government encouraged development of nearly 500 high-tech agricultural cooperatives (BritCham Vietnam, 2021) to help individual farmers grow strong together via cooperation through a professional, supportive association. This signifies forthcoming policy opportunities for PA adoption.

### Factors Affecting Behavioural Intention to Adopt PA Technology

In the PA literature, conceptual adoption models – Technology Acceptance Model (TAM), Theory of Planned Behaviour (TPB), and Diffusion of Innovation theory (DOI) – have commonly helped identify factors underlying behavioural intention to adopt technologies. However, these models do not account for all factors in the complexity of users’ behavioural intention. Vietnamese farmers are the only decision-makers on their farms (World Bank Group, 2016). Hence, Salimi et al. (2021) suggested that agricultural innovation adoption research should also consider farmer characteristics as primary decision-makers. We therefore adopt the Unified Theory of Acceptance and Use of Technology (UTAUT), as the most comprehensive model to account for the complexity of behavioural intention and the effects of different contextual factors on that intention (Venkatesh & Bala, 2008).

**Figure 1.**
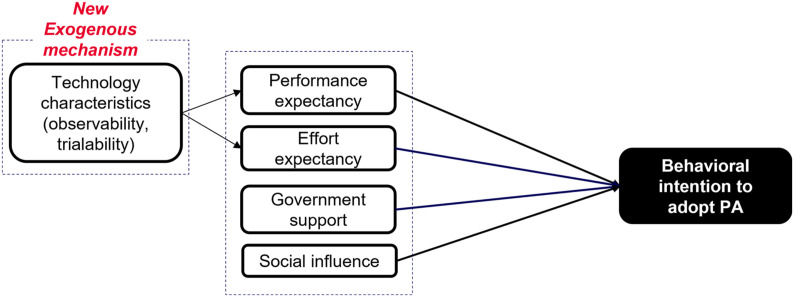
Conceptual framework adopted by this study building on the UTAUT model and its extension to the exogenous mechanism. (Source: Authors adapted from Venkatesh et al., 2016).

We focus on intention as the only dependent variable to represent farmer willingness to adopt PA technologies in the future. The conceptual framework guiding data collection and analysis is presented in Figure 1, drawing on UTAUT and proposed extension to the exogenous mechanism with trialability and observability. UTAUT extensions suggest a variety of possible additions to the original model (Venkatesh et al., 2016), which become this study’s theoretical contributions.

In Figure 1, the construct behavioural intention (BI) refers to motivational factors that influence technology-adopting behaviour: the stronger the intention, the more likely the adopting behaviour (Sun & Gao, 2020). Venkatesh et al. (2003) and Li et al. (2020) proposed four main UTAUT constructs: performance expectancy (PE) and effort expectancy (EE) as technology attributes, social influence (SI), and government support (GS) as contextual factors.

PE indicates the extent to which an individual perceives that using technology will help achieve gains in job performance (Onaolapo & Oyewole, 2018). PE is important for adoption because benefits and utility in improving performance are influencing factors in user’s intention to adopt (Castro & Hernandez, 2019; Lutfie & Marcelino, 2020; Syifa & Tohang, 2020). PE pertains to constructs of other models: the perceived usefulness of TAM, TAM2, TPB, the relative advantage of DOI, and outcome expectation of Social Cognitive Theory (SCT) (Venkatesh et al., 2003). PE and similar constructs significantly impact behavioural intention (Davis, 1993; Venkatesh et al., 2003; Venkatesh & Morris, 2000). In PA adoption, PE may refer to reducing production costs, increasing yield, efficient farm management, and protecting the environment (Adrian et al., 2005; Li et al., 2020).

Effort expectancy (EE) is the degree of ease associated with using the technology (Venkatesh et al., 2016), with the similar notion to perceived ease of use in TAM and TAM2 and complexity in DOI theory. Similarities, including measurement scale and definitions, have been previously recognized (Davis et al., 1989; Moore & Benbasat, 1991; Venkatesh et al., 2003). Studies have confirmed that EE significantly influences behavioural intention to use technology and is especially significant for first-time and trial users (Christopher et al., 2001; Min et al., 2008; Venkatesh et al., 2012).

Social influence (SI) measures the degree to which an individual perceives that others believe that they should use the technology (Venkatesh et al., 2016). This was previously addressed as the social norm in the Theory of Reasoned Actions (TRA), TPB, or image in DOI theory (Venkatesh et al., 2003). SI implies that an individual’s intention is influenced by the effect of other people’s opinions on whether they should use the technology. SI has an important role at adoption’s beginning, potentially affecting behavioural attitudes (Omar et al., 2021; Swinerd & McNaught, 2015). Li et al. (2020) tested this hypothesis, too, and found that the way communities, peer groups, and other social influences encourage farmers’ use of PA technology affected their behaviour, and possibly attitudes.

Being a socialist market-oriented economy, Vietnam government plays a different role and has different effects on people’s behaviours than in other countries (Chong et al., 2010). It has a controlling role in the economic system and operations of many organizations. Critically, it influences the establishment of facilitating conditions to remove technology adoption barriers (Zhou et al., 2010). Li et al. (2020) argue that facilitation conditions have a stronger effect than other determinants of PA adoption intention: access to government-provided financial support and consulting services are considered proxies of facilitating conditions. Here, instead of using facilitating conditions, we proposed to examine the influence of government support on farmers intention to adopt PA technology.

This research proposes the potential effects of technology’s trialability and observability on PE and EE as a new theoretical contribution to the original UTAUT; these have been underexplored in the existing literature. Rogers (2010) theorized trialability and observability as two of five constructs explaining technology acceptance and adoption in DOI theory.

Trialability, or the degree to which potential adopters can explore the technology, is said to manifest in a positive exploratory experience, which can foster adoption (Shang et al., 2020). Rogers (1983) theorized that user intention to adopt technologies depends on the extent to which potential adopters have no-obligation opportunities to experiment with the technology. Experimentation helps potential adopters assess the importance of pre-use information, which can reduce ambiguity and uncertainty about the new technology (Oluyinka et al., 2021). Other studies revealed that the ability to trial innovations allows potential users to realize benefits, minimize presumed difficulty and gain sufficient experience on effective technology use (Almaiah & Alyoussef, 2019; Mouakket, 2020; Tchetchik et al., 2020). This implies a strong connection between trialability and users’ PE and EE. In information system studies, trialability is an important variable for investigation in technology-acceptance research. In agricultural innovation research, the importance of facilitating farmers’ initial exposure and ease of experimenting new technologies is confirmed, especially for those requiring high initial investment (Yigezu et al., 2018).

Observability is the extent to which the benefit of using the technology is visible to potential adopters (Rogers, 2010). The more easily potential users can witness the advantages, the higher the probability of acceptance (Anjum, 2019). In information system research, studies have redefined technology’s observability by two independent constructs: visibility and result demonstrability (Naglis & Bhatiasevi, 2019; Yuen et al., 2021). Visibility is the degree to which potential users can clearly see and hear about the innovation from multiple communication channels, including television, the newspaper, and the internet (Naglis & Bhatiasevi, 2019). On the other, the resultant demonstrability may be viewed in terms of technology’s positive outcomes being clearly demonstrated to potential adopters (Yuen et al., 2021).

## Methodology

### Data Collection

This study was based on a sample of 35 participants, including 25 rice farm owners and 10 experts in the fields of agronomy, agricultural engineering, mechanization, and agricultural innovation policy. Details of participants and selection criteria are presented in Table 1. To ensure that the study included appropriate experts, the research team identified potential participants based on their level of involvement and contributions to Vietnam’s agricultural innovation. Public media platforms were utilized to identify potential interviewees. The supporting companies of this project were also involved in identifying interviewees; their expertise in agricultural innovation and their industry networks were valuable resources in identifying suitable participants. Subsequently, a filtering for engagement level in innovation refined the list of experts. The specific criteria for the filtering process are presented in Table 1 for transparency and clarity.

**Table 1.** Participants and Selection Criteria.

Group of participants	Number of interviews	Subgroups of participants	File codes	Selection criteria
Agricultural activists, project leaders	4	Leader of agricultural innovation projects	AE01	>10 years’ experience in agriculture
		Expert in agricultural sustainability	AE02	
		Experienced specialist in rural development	AE03	
		Experienced specialist in rural development	AE04	
Technology developers	3	Agriculture technology developers and vendors	TD01	>10 years’ market experience
			TD02	
			TD03	
Representative of the local authorities	3	Department of agriculture of a northern province	LA01	>10 years’ experience working with farmers; directly involved in rice farming administration
		Department of agriculture of a central province	LA02	
		Department of agriculture of a southern province	LA03	
Farmers in the South	11	Rice farmers in Long an province	FO01	Rice farming with >1 ha and <2 ha of arable land
			FO02	
			FO03	
			FO04	
			FO05	
			FO06	
		Rice farmers in Dong Thap province	FO07	
			FO08	
			FO09	
		Rice farmers in An Giang province	FO10	
			FO11	
Farmers in the central	7	Rice farmers in Quang Ngai province	FO12	
			FO13	
		Rice farmers in Phu Yen province	FO14	
			FO15	
			FO16	
		Rice farmers in Binh Dinh province	FO17	
			FO18	
Farmers in the North	7	Rice farmers in Thai Binh province	FO19	
			FO20	
		Rice farmers in Hung Yen province	FO21	
			FO22	
		Rice farmers in Thanh Hoa province	FO23	
			FO24	
		Rice farmers in Ha Nam province	FO25	
**Total**	**35**			

**Figure 2.**
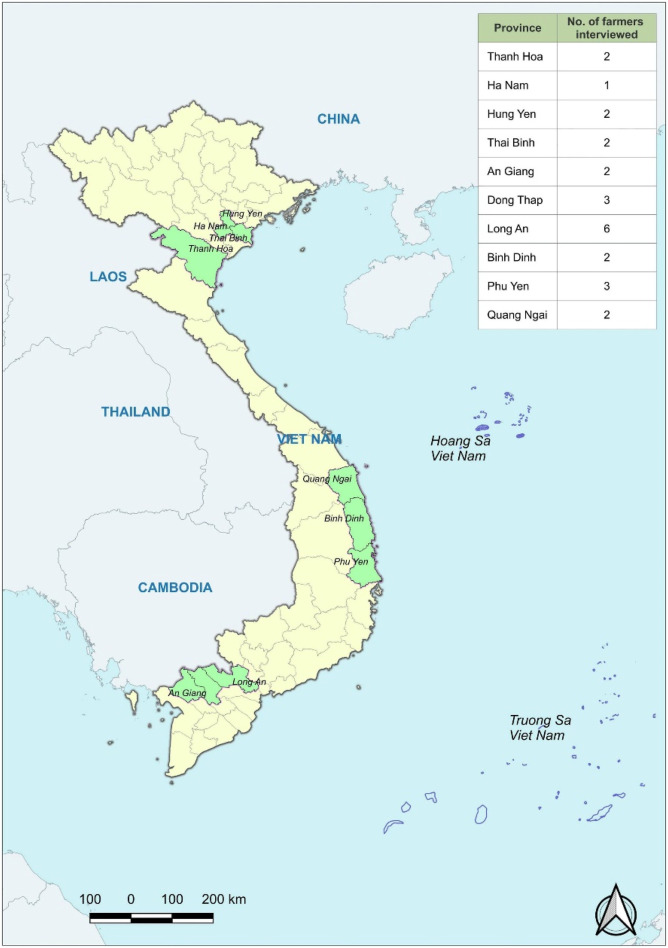
Geographical distribution of interviewed farmers (created using QGIS).

For rice farmer participants, we used purposeful sampling approach (Patton, 1990) and obtained variation in the sample by strategically selecting cases across regions of Vietnam (Lincoln & Guba, 1985). Stratified purposeful sampling involves selecting categories of individuals to include in the final sample. The sample is then divided into strata based on the participant characteristics, and a specific number of participants are assigned to each stratum (Suri, 2011). This study categorized farmer participants by geographic differences, enabling the researchers to gain insights from across Vietnam (Figure 2).

The farmers were identified in a sequential process. First, we interviewed ten experts and used interview notes to create preliminary themes for guiding farmer interviews. As we progressed, we asked the expert participants to suggest farmers from their network who could provide additional insights to improve and expand upon the preliminary themes. The two supporting companies helped contact the recommended farmers and invited them to participate in our interview sessions. Farmer selection was also designed to ensure a sample across regions of Vietnam.

Semi-structured interviews were considered the most appropriate method to investigate in-depth farmer perceptions of future PA adoption. The interview guide was developed and structured around the constructs of the UTAUT model. Participants were given opportunities to explain and build on their responses, as it is crucial for the researchers to understand the meanings that farmers and experts ascribe to the phenomenon of PA adoption. Participants have the autonomy to extend their thinking to other areas that the researcher has not considered but are significant in enhancing the understanding of the phenomenon (Saunders et al., 2016). The Interview guide’s structure and key questions are presented in Box 1.

**Box 1: Interview Guide**

**Section A: General Questions About Agricultural Innovation in Vietnam to ‘Warm Up’**

**Section B: Key Questions About the Factors Influencing the Behavioural Intention to Adopt PA**

• Regarding the technology itself, what are the important attributes that would make the technology accepted and adopted by Vietnamese farmers? (Probe where necessary: performance expectancy, effort expectancy, observability, trialability, and associated risk).
• Has the communication of precision agriculture technology effectively reached rice farmers? To what extent is it widespread among peer farmers? What is your perspective on the effectiveness of this communication?
• Regarding the nature of rice farms in Vietnam, what are the important farm characteristics that may affect PA adoption? (Probe where necessary: Farm size, resource availability)
• How might traditional farming practices affect the future adoption of PA technology?
• What do you think about the impacts of external facilitating conditions (e.g. linkage, external supports) on PA adoption in Vietnam? Are those conditions accessible to all farmers?

**Section C: Concluding Question**

• Do you think that Vietnamese smallholder rice farmers have the potential to facilitate a wider adoption of PA technologies?

Interviews adhered to the ethical procedure as approved: questions were designed to not distress participants and were audio-recorded with participants’ permission. No personal information was collected that may identify participants. All interviews were conducted from January 3^rd^, 2021, to May 13^th^, 2021, ranging between 35 and 50 minutes.

### Data Analysis

**Figure 3.**
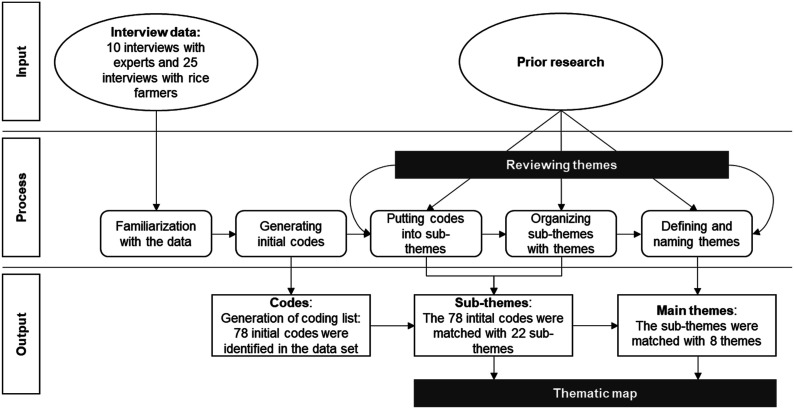
Iterative thematic analysis approach (adopted from Matt et al., 2019; Becker et al., 2017; Braun & Clarke, 2019).

To generate insights from the qualitative data collected, we adopt the rigorous thematic analysis approach (see Figure 3). For Braun and Clarke (2006), thematic analysis and its flexibility allow researchers to play an active role in determining how exactly the analysis will be used to ensure an overall fit in the research design (see also Saunders et al., 2016). Besides, the iterative process of thematic analysis adopted by this study enables matching of interview codes, sub-themes, and themes through constantly reviewing the literature. Iterative analysis allows researchers to alternate between considering existing theories and emergent qualitative data (Tracy, 2019). Figure 3 details the approach, which is not a linear phase-to-phase process but a recursive cycle, allowing the research team to move back and forth between different phases.

Interviews were transcribed, then translated from Vietnamese to English, and then were repeatedly read and reviewed to ensure translation consistency. Initial codes were generated by searching for recurring data patterns, with 78 codes identified in the data set. Codes were merged into 22 sub-themes, which were then allocated to eight main themes (six themes in the proposed model and two newly emerging themes from the data). The whole process was powered by NVivo 12 Pro. We analysed the transcripts and coded them manually to ensure that issues associated with term variations, plurality or singularity, and ways of expression were carefully accounted for.

To ensure the trustworthiness of the analysis process, intra-coder reliability was seriously considered. This refers to consistency between coding decisions made by a single coder or researcher in qualitative research (Saldaña, 2021). Developing a clear coding scheme is important for addressing intra-coder reliability in qualitative research (Saunders et al., 2016). This involves creating a concise, comprehensive coding scheme that includes all relevant themes and categories. The literature review of this study suggested a coding scheme to set guidelines for systematically identifying, labelling, and categorizing data. The coder also improved intra-coder reliability by regularly checking and rechecking codes (Saunders et al., 2016), involving revisiting codes already assigned to a portion of the data and verifying their continued accuracy (Saldaña, 2021). By doing so, the researchers were enabled to identify any discrepancies in the coding and address them before they became more problematic (Tracy, 2019).

Constant review of the literature accompanied the process of matching codes with sub-themes and themes. This allows for exploring and defining newly emerging themes: such themes, outside of the proposed conceptual model, are welcomed, if the research team could clearly define them in relation to existing theories. This aligns with the advantage of the iterative thematic analysis approach, which allows the combined use of deductive and inductive methods (Braun & Clarke, 2021; Tracy, 2019). As the last analytical step, themes and sub-themes were defined, and a thematic map was created.

**Figure 4.**
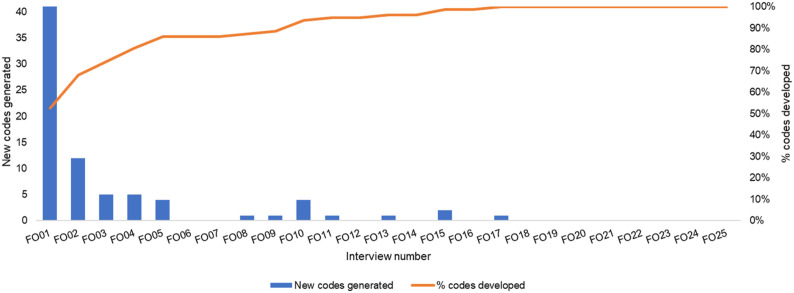
Timing of code development.

To provide evidence for data saturation, Hennink and Kaiser (2022) suggested code frequency counts, which involves scrutinizing every interview or focus group transcript and enumerating the number of fresh codes in each succeeding transcript or transcript group until the incidence of new codes starts to diminish, indicating that saturation has been achieved (Ando et al., 2014; Morse et al., 2014). Figure 4 shows empirical evidence of data saturation by specifying the number of new codes generated and the accumulated percentage of code developed from each subsequent interview.

Clearly, farmers have been actively engaged in code development. As we move towards the series end, the number of new codes generated decreases, which suggests that data saturation may have been reached, most relevant codes have already been generated, and further efforts may yield diminishing returns (Hennink & Kaiser, 2022; Namey et al., 2016).

We used a five-level scale to aid the presentation and visualization of qualitative data (Specht et al., 2016). The assessing levels ranged from ‘very important’ (**+++++**) to (**+**) ‘not significant’ to assess each sub-theme’s significance within the themes. The main criterion was the frequency of arguments in the interviews (Table 2). This assignment is qualitative and is not derived from substantial quantitative analysis. This scale only illustrates the descriptive part of the findings.

**Table 2.** Explanation of the Five-level Scale (Source: Adapted From Specht et al., 2016).

Importance	Scale	Criteria Participants citing this idea
**Very important**	**+++++**	Over 20 participants
**Important**	**++++**	16–20 participants
**Moderately important**	**+++**	11–15 participants
**Slightly important**	**++**	6–10 participants
**Not important**	**+**	0–5 participants

## Results

**Figure 5.**
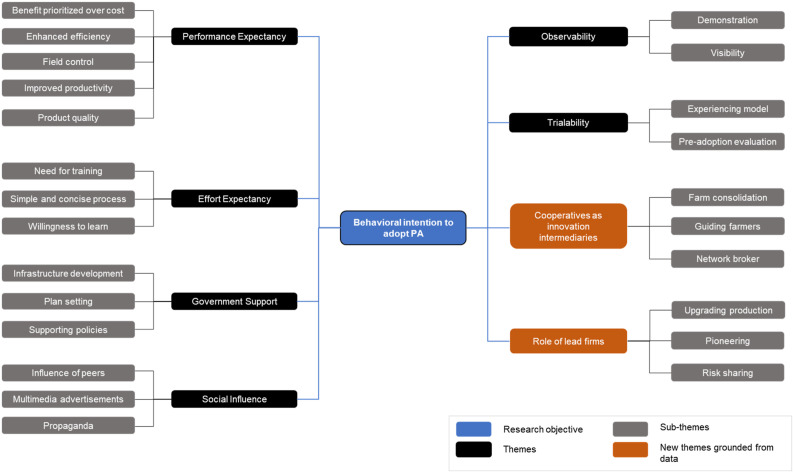
Thematic map of themes and sub-themes.

Our results are synthesized in Table 3, and the thematic map is used to visualize them (Figure 5). The map contains eight themes (performance expectancy, effort expectancy, government support, social influence, observability, trialability, the performance of cooperatives as innovation intermediaries, and the role of lead firms) and 22 sub-themes. Each theme and sub-theme were defined and analysed with verbatim quotes from interviews.

**Table 3.** Themes and Sub-themes Related to the Empirical Analysis of PA Adoption in Vietnam.

Theme/Sub-theme	Importance	Mentioned by no. of participants
Performance expectancy		
Benefit prioritized over cost	**+++**	14
Enhanced efficiency	**+++++**	21
Field control	**+++**	12
Improved productivity	**+++**	13
Product quality	**++**	10
Effort expectancy		
Need for training	**++**	7
Simple and concise process	**++++**	20
Willingness to learn	**++++**	16
Government support		
Infrastructure development	**++**	6
Plan setting	**+**	4
Supporting policies	**+++**	13
Social influence		
Influence of peers	**++**	10
Multimedia advertisements	**++**	8
Propaganda	**+**	2

Theme/Sub-theme	Importance	Cited by no. of participants
Observability		
Demonstration	**++++**	17
Visibility	**++++**	20
**Trialability**		
Experiencing model	**+**	5
Pre-adoption evaluation	**+**	2
Cooperatives as innovation intermediaries		
Farm consolidation	**+++++**	32
Guiding farmers	**+++**	11
Network broker	**+++++**	31
Role of lead firms		
Upgrading production	**++**	7
Pioneering	**+**	4
Risk sharing	**+**	4

*Results are presented using a five-level scale ranging from very important (+++++) to least important (+).

To identify relevant findings, we conducted a thorough analysis of our qualitative data using in-vivo coding, which involved assigning codes to each quote based on the words or phrases used by participants. We then used the constant comparative method (Glaser, 1965) to identify patterns in the data and assign the codes to themes and sub-themes. For example, one sub-theme identified under ‘Performance expectancy’ was ‘Field control’, supported by one quote: ‘To reduce fertilizer usage, it is essential to monitor both the living conditions of crops and the soil conditions to have better sense of control over the field’. This was coded under ‘Field control’, because it relates to the participant’s desire to have better control over agricultural practices. We then categorized quotes related to ‘Field control’ under ‘Performance expectancy’, because they reflect the participant’s desire to have control over agricultural practices, which is linked to their PE. This is supported by the conceptualization of PE by Venkatesh et al. (2003). Similarly, other quotes were categorized into sub-themes and themes based on their relevance (see Table S1 of the Supplementary Material for the data structure with themes, sub-themes, and representative in-vivo codes).

Throughout our analysis, we used terms specific to agriculture, such as ‘soil conditions’ and ‘agricultural cooperatives’. To ensure our findings are accessible to a wider audience, we have included definitions of these terms.

### Main themes of the Proposed UTAUT Extension

#### Performance Expectancy (PE)

PE represents the extent to which farmers perceive that using PA technology will help them achieve farm management efficiency and effectiveness or PA technology’s potential benefits (Li et al., 2020). The researchers found that PE received the most participant attention, with a considerable superiority in number of codes and sub-themes compared to other main themes. More specifically, enhanced efficiency, field control, improved productivity, product safety, and quality are categorized as sub-themes under PE (Table 3).

Farmers tend to prioritize benefits expected from the technology over considering initial investment costs. Most farmers and agricultural experts argued that, when the technology could solve the current pain points in rice farming, the high initial investment would be affordable through different financing channels (FO04, FO22, and LAO03). Farmers also showed their awareness of cost and benefit evaluation when considering adopting the technology: they recognized that they ‘will get what they pay for’ (FO14). One agricultural expert claimed:

I think the initial investment is not the point. When a person is farming, they must buy equipment to serve their farming. Like when they bought a plow mower, they now invest in high-tech agricultural practices to save labor, precisely apply inputs, and save the environment. If the technology is effective, the farmers will invest. (AE01)

Of sub-themes examined, enhanced efficiency is the most addressed category, with 21 of 35 participants relating their discussion to input efficiency. Participants emphasized the need to apply inputs (water, fertilizer, and pesticide) more efficiently. The arguments to clarify the significance of enhanced efficiency are twofold. First, recent dramatic increases in fertilizer prices have hindered rice-farming profitability, which ‘created difficulties’ (FO12), ‘discouraged farmers’ (FO23), and some even ‘intended to abandon their rice fields’ (FO25). This leads to the need for tools that help save fertilizer or enable more efficient fertilizer use. Second, following their ancestors’ farming practice, well-embedded in tradition, farmers often overuse chemicals. One agricultural expert said, ‘instead of applying 1 kg as recommended by fertilizer manufacturers, they apply 4.5 kg; the excess fertilizer will be evaporated or washed away by water’ (TD01). Farmer interviewee revealed they expected PA technology could help them measure and apply chemicals precisely to enhance efficiency. One said:

We often fertilize according to personal experience, so sometimes the plants are also over-nutrient, which also generates fertilizer waste. If there is a technology that can know exactly how much the plant is deficient or excessive to fertilize, it will greatly increase efficiency and save cost. (FO25)

In field control, participants perceived the ability to understand the needs of plants and keeping track of soil conditions as expected benefits of PA. Soil conditions refer to the physical, chemical, and biological properties of soil, such as texture, nutrient content, pH, and microbial activity (Zhao et al., 2020). Farmers especially showed enthusiasm about a solution that could help them calculate the exact amount of nutrients needed by the plants. This gives them a sense of control over the whole process of applying chemicals. A participant said that, to understand plants better, farmers expect PA technology to help them ‘listen to the plants’ (TD03). Participants also stressed the need for monitoring soil conditions (AE02, FO12): keeping track of plant living conditions would accompany understanding of plants and provide the most optimal reference of input level:

The technology I need the most is the one that helps farmers measure the amount of nutrients that a plant needs so that farmers can fertilize accurately, help rice bloom better, develop evenly, and be healthier. It should also help farmers save fertilizer and avoid waste. (FO11)

From the perspective of productivity and product quality, participants expected that PA technology could help farmers achieve a balance between increased productivity and enhanced product quality. As the agricultural experts argued, for years, agriculture practice of Vietnam had been planned and directed towards high productivity (LA01, AE03), but the ‘stability of quality and safety of products have not attracted much attention’ (AE01). With a sole focus on enhancing farming productivity, rice farmers have abused fertilizers and pesticides and ignored the chemical residues and side effects of chemicals on the environment. A representative of the local authority, stated:

Farmers think the rice will grow better when applying more fertilizer, and the field will get a higher yield. But they are unaware that applying too much fertilizer will lead to saturation while having no effect. This trend happens not only with rice farmers but in all fields. (LAO03)

Many participants held that Vietnam’s agricultural products had not met standards of strict markets such as Europe and the US regarding chemical residue compliance. They were looking for technologies that could help regulate the application of chemicals in an optimal and precise manner to control residues and better comply with agricultural product safety standards. This is expected to add more value to Vietnam’s agricultural products to compete in high-expectation markets. One agricultural expert argued:

If we want to enter big markets, our products must be clean, nice and exceptional, and to do so, we must apply and operate irrigation and cultivation technologies following the expected standards of those markets. And we must be able to monitor and control the whole process. (AE01)

#### Effort Expectancy (EE)

EE explains the degree of ease associated with using PA technology as perceived by farmers (Lin & Lo, 2015). This theme is built up from three sub-themes: participants’ discussion on the need for training, their expectation of a simple, concise process when using PA technology, and farmers’ willingness to learn (Table 3).

Of the sub-themes, participants highlighted the need for a simple, concise process of using PA technology. Specifically, 20 were concerned about the technical difficulties they might face when applying the technology. Some farmers even resisted using technologies, because they thought that applying advanced technologies was ‘cumbersome and troublesome’ (FO03, FO17). The complexity of using technologies thus appeared to be the biggest hurdle that prevents farmers from advancing in agricultural innovation (FO19). One participant suggested that the technology should be simplified to fit farmers’ level and knowledge:

The technology should be easy to use, because farmers’ knowledge of advanced technologies is not high compared to people in other industrial sectors. (FO22)

We also found that training on how to use PA technology is essential in helping farmers become confident in their future adoption. To farmers, training is ‘hands-on training sessions’ (FO17) and ‘constant consultation’ with technology experts (FO12). Training sessions could be organized by technology suppliers or the local authority at ‘different levels’ (provincial, district, and commune) to ensure sufficient exposure to most farmers (TD01). A farmer participant claimed that, if training was well articulated, trained farmers would ‘communicate and instruct more peer farmers’ (FO19). This enhances the awareness of PA among farmers. Also, frequent consultation with experts or the vendor’s technical support teams could make farmers feel ‘secure, comfortable, and confident’ (LAO02) in their ability to apply complicated technologies, including PA. Another agricultural expert affirmed:

Farmers cannot apply the technology by themselves. They should have a consultant by their side, supporting them, so that they can apply to work and learn simultaneously. (AE02)

The willingness to learn refers to the degree to which farmers are open to and enjoy seeking new experiences with innovation. We found that Vietnamese rice farmers were relatively open to learning new things, as they perceived that they need to ‘catch up with the technology era’ (FO15). If the technology is proven effective in helping farmers improve farming practice, they are willing to learn and overcome technical complexities (TD02). Regardless of educational levels, farmer participants showed their innovativeness and curiosity about the advanced solutions that can ease their current pain points in farming:

Although I am just a farmer with a low level of education, I am open to receiving new things. Since a young age, I have loved to discover things. (FO21)

#### Government Support (GS)

Government support includes initiatives by government as a mechanism to create facilitating conditions for the future adoption of PA technology (Bukchin & Kerret, 2020). Government support comprises three sub-themes (Table 3). In general, we found that farmers expected a strong government involvement in transforming and upgrading current farming practice.

The first sub-theme, infrastructure development, was viewed by participants as government investment in roads, water supply systems, drainage, and electricity grids to accommodate adoption of advanced technologies (LAO02). Since agricultural land in Vietnam varies with a complex topographical nature, characterized by ‘many hills, mountains, and slopes’ (FO24), it has challenged the modernization of farming activities on a large scale. Consequently, it is crucial for the government to invest in infrastructure to facilitate and ensure the feasibility of technology adoption. Some participants shared that their farming plots ‘were positioned in remote areas’ (FO07, FO15), making them hard to reach. This suggests improvements in infrastructure (e.g. roads) that enable better accessibility of currently isolated farmers to technology introduction, consultation, demonstration, and promotion.

The other two sub-themes, plan setting and supporting policies, constitute two major proxies of government support. Plan setting means the ‘exclusive initiative taken by the government’ (FO19) to sketch roadmaps and guidelines for stakeholders involved in agricultural innovation. A well-established plan was claimed to strengthen collaboration among stakeholders (farmers, technology developers, businesses, and the government) by clearly ‘stating the role of and expectation from each stakeholder group’ (FO22). Thirteen said that the mechanism of supporting policies was significant in determining farmers’ behavioural intention to adopt PA technology. Farmers expected policies to focus on ‘capital support’ (FO08, FO15) and ‘subsidies’ ((FO19); agricultural experts were more inclined towards policies focused on programs supporting technology transfer (training, consulting, and directing) (LAO01, LAO02, and TD01). Although the government has initiated policies to support agricultural innovation, some farmers were had gained little benefit. It is thus essential for policymakers to rethink how to ensure sufficient exposure of supporting policies to all farmers. One farmer claimed:

Supporting initiatives, notably financial subsidies, only go to fields owned by the state and corporations, not individual smallholder farmers like us. We face lots of challenges in gaining access to these supporting packages. (FO20)

#### Social Influences (SI)

SI implies that intention is influenced by other people’s opinions whether they should use the technology (Omar et al., 2021). SI has three sub-themes: influence of peers, multimedia advertisements, and propaganda. Generally, farmers felt optimistic about PA when exposed to opinions supporting technology adoption.

Influence of peers had a significant impact on intention to adopt PA. Farmers showed willingness to adopt when other farmers in the community had applied it and had positive feedback because farmers tended to fear lagging behind their peers: the ‘gap between farmers would be widened’ (FO04, AE03) if the opportunity to apply advanced technology was only accessible to some farmers. Also, if farmers found that peers applied the technology effectively, they would be inclined to use it (FO03), due to existing good bonds and trust in farmer communities:

In Vietnam, farmers have a spirit of mutual affection, love, and respect for neighbours. When one person needs something, others are ready to share and help. (AE02)

Participants viewed multimedia advertisements as ineffective in convincing farmers to adopt the technology – they were viewed as only significant for increasing people’s awareness. Farmers seemed to prefer ‘hands-on experience’ (TD01) and ‘directly observing’ (FO04) actual benefits of PA technology than believing what was ‘propagated on newspapers or TV shows’ (FO18). Some participants saw technology advertisements and promotion programs by government as ‘propaganda’ (FO18, AE02), by which they implied a skeptical attitude toward communicated messages. Participants described ‘propaganda’ as information of a biased or misleading nature, communicated to publicize a particular viewpoint – a ‘powerful tool to spread the awareness’ of PA technology (AE01), but some showed hesitation on whether to follow the message or not. One participant said:

Farmers declined to adhere to any propaganda line. They may not understand the message. However, propaganda increases conservatism, which makes farmers deliberately not understand. (AE02)

Despite showing no clear effect on farmers’ behavioural intention to adopt, advertisements are still considered important due to their perceived significant impacts on increasing PA technology awareness.

#### Observability and Trialability

Alongside the main themes under the UTAUT framework, we also assessed the two extended themes following the DOI theory of Rogers (2010): observability and trialability. First, observability is the extent to which PA technology benefits are visible to Vietnamese rice farmers. Observability consists of demonstration and visibility (Table 3). Farmers were generally more confident in the future application of the technology when promised benefits and advantages were visible and proven. Demonstration is described by the participants as exhibitions explaining how the technology is implemented to benefit farmers. Experts and farmers agreed that demonstration in the sample field was a prerequisite for future adoption. Farmers tended to perceive what they heard from promoters of PA technology as ‘theories’ (AE02, FO13, and FO24). As a result, demonstration to show farmers how PA technology outperforms their traditional farming methods is crucial in determining intention to adopt:

Farmers think advanced technologies are just theories until they can see that if the technology is applied, it actually improves the performance. (AE02)

However, technology developers should commit to the technical attributes that they have showcased. Gaining the trust of farmers takes a lot of work. If farmers realized shortfalls in the applied technology compared to what they had been shown, they would never ‘give it another chance’ (FO19). We found that farmers faced many risks when investing in technology. The most considerable risk mentioned by our participants was the ‘financial risk’ they would face when they had to borrow money from institutions to invest in such an expensive technology (FO21, FO22, FO24). If the technology ‘fell behind expectation’, there would be a big loss for farmers, especially the smallholders (FO11). This means that, despite good demonstrations, technology vendors should be aware of their commitment to avoid disappointing farmers.

Visibility is the extent to which farmers can directly observe others applying PA technology. Although visibility also aims to increase awareness of PA technology, it differs from advertisements and propaganda (Section 4.1.4). Visibility here implies ‘farmers’ experience with the technology through seeing others in their area benefit from that technology’ (FO15), whereas advertisements and propaganda are others’ words which farmers do not trust. We found that technology developers would need more than just a demonstration to convince farmers and make technology’s benefits ‘really visible’. Farmers need to see ‘detailed but straightforward calculations and presentation’ (FO16, AE01) of the benefits before adopting the technology:

You must deploy some farmers to try it first, so other farmers around will see if it's effective, then they will decide whether to use it or not. (FO11)

Technology’s trialability is like observability in that it provides potential adopters of PA technology with initial experiences before official adoption. However, trialability refers to the degree to which potential adopters can explore the technology by themselves rather than observing others applying it. The interviews did not reveal much attention paid to trialability of PA technology, because farmers did not expect technology vendors to let them try ‘such an expensive technology for free’ (AE01). Yet, through a deeper investigation, we found two sub-themes under trialability: the experiencing model and pre-adoption evaluation (Table 3).

Participants described the experiencing model as sample pieces of technology provided by technology vendors to give farmers opportunities to ‘experiment’ with the technology ‘without any obligation’ (AE01, FO22, LAO03). Experimentation with the technology sample was found to influence farmers’ behavioural intentions considerably. One participant stated that ‘sufficient trial time’ would help reduce the ‘ambiguity and uncertainty’ related to the innovation implementation (TD01). Two technology vendors believed that they could tolerate a 6-month to 1-year trial period for farmers (TD01, TD02).

The success of PA-experiencing models is the foundation for positive pre-adoption evaluation of farmers, another sub-theme of trialability. This can be defined as when trial innovations allow potential users to ‘realize the benefits’ (AE01), ‘minimize any presumed difficulty’ (FO22), and ‘gain sufficient experience’ (LAO03) on how to use the technology effectively (Almaiah & Alyoussef, 2019; Mouakket, 2020; Tchetchik et al., 2020). From the interview with a farmer, we identified one good practice to ensure the trialability of PA technology for farmers: PA trials could be provided through the support of ‘agricultural extensions’ (FO22), also known as local agricultural advisory services.

### Newly Emerging Themes

From the data, building on the themes under the proposed extended UTAUT model, we identified additional themes which have not yet been categorized. Therefore, following an iterative process of welcoming new themes from collected data and making sense of them through constantly reviewing the literature (Tracy, 2019), we found that theories about the innovation-leading role of agricultural cooperatives and the support of lead firms could explain and accommodate the uncategorized discussions from participants. To analyse these two emerging themes, we first begin with the definition of their sub-themes, then identify appropriate theories to explain the importance of each theme.

#### Performance of Agricultural Cooperatives as Innovation Intermediaries

The interviews with both expert and farmer participants focused on the role of agricultural cooperatives. We have synthesized these cooperative-related discussions with three outstanding sub-themes under the main theme of agricultural cooperative performance: farm consolidation, guiding farmers, and network brokers (Table 3). These sub-themes generally describe how farmers perceived the effectiveness of their local agricultural cooperatives in supporting and facilitating the adoption of PA technologies.

Farm consolidation means a planned readjustment and rearrangement of fragmented land parcels owned by individual farmers to form larger fields under the management and control of one organization representing the whole group of land contributors (Nguyen & Warr, 2020). The participants seemingly supported the idea of putting small plots together to form consolidated areas for farming under one unified management scheme. They knew that advanced technology, including PA, could be infeasible in fragmented fields. The fragmented and small-scale farms were considered hurdles for technology applications in terms of ‘low return on investment’ (FO05) and ‘limited resource availability’ (FO22). As perceived by our agricultural experts, the required farm size should be from three to five ha to make PA adoption feasible (LAO03, TD02), while the average size of rice fields in Vietnam is 1.2 ha (FAO, 2018). One expert commented:

I think small-scale is the most important hurdle. If we want to apply precision technology, we must gather small fields together into one place; it will be easier to control and apply. (AE04)

This is where agricultural cooperatives execute their ‘major role in linking farmers’ (AE01, LAO02, FO14) to form a strong farming ally with bigger farm sizes and more resources for technology investment. Farmers are vulnerable and have always suffered from the external environment, such as weather and market competition. Therefore, they tend to cooperate or become a part of either an organization or an association to become stronger (Altman et al., 2020).

Under another important sub-theme, network broker, participants identified cooperatives as boundary organizations to help farmer members increase exposure to support from the government and businesses. De Silva et al. (2018) defined network brokers as farmers’ representatives to fulfil their liaison position with the many actors in the technology adoption process. From the farmer participants’ perspective, despite various support packages initiated by the government to foster technology adoption, smallholder farmers needed help gaining access to those packages. They suspected that these packages went through agricultural cooperatives as intermediaries before reaching individual farmers rather than supporting smallholder farmers as direct beneficiaries:

The government never provides direct capital support to farmers; they are often through to the cooperatives, agricultural extension societies or businesses. (FO15)

Furthermore, instead of directly contacting each farmer, which may take time and effort, corporates could use agricultural cooperatives as their contact point to spread their support to farmers (LAO02) in a ‘faster and more convenient way’ (FO15).

The guiding farmers sub-theme received the least attention from participants. Yet it is controversial, since it has attracted opposing views from farmer and expert participants. Theoretically, guiding farmers is described as one of the cooperative’s major roles in introducing the technology to farmers and supporting them in the adoption (Yang et al., 2014). Agricultural experts believed that cooperatives had been key players in guiding and supporting farmers with technology applications (AE02, LAO03), but farmers perceived the performance of agricultural cooperatives as inefficient. Some farmers even contended that the establishment of cooperatives was ‘just a formality’ (FO08). However, despite continuing arguments, cooperatives were still considered a potential innovation hub to support farmers in technology adoption if they were well managed by ‘talented leaders’ (FO01). An expert participant said:

The performance of cooperatives has good and bad sides, but in theory and practice, we need to recognize the potential role of cooperatives in helping farmers get exposed to, transfer, and maintain technology adoption. (LAO02)

In sum, all three sub-themes, farm consolidation, guiding farmers, and network broker, have emphasized agricultural cooperatives’ significant role in facilitating PA adoption. These sub-themes can be summarized under one main theme following the theory of innovation intermediary by De Silva et al. (2018), which highlights the key roles of innovation intermediaries in supporting innovation processes, notably knowledge intermediation and innovation intermediation. In agriculture, innovation intermediation can be explained by discussion of farm consolidation and network brokers, while knowledge intermediation is more relevant to the sub-theme of guiding farmers (Yang et al., 2014). As such, the performance of agricultural cooperatives as innovation intermediaries is appropriate as the main theme that generally captures the content in this section.

#### The Role of Lead Firms

Another theoretical contribution of this study is the role of lead firms in boosting PA adoption. From the literature on value chain management and the purpose of this study, lead firms can be defined as firms of various sizes that have backward commercial linkage with rice farmers (i.e. buying products from farmers) (Clay & Feeney, 2019). Lead firms may include buyers, traders, exporters, and processors (De Marchi et al., 2019). From interviews with our participants, lead firms were expected to play a crucial role in facilitating PA adoption by helping farmers upgrade their production, pioneering in technology advancement, and sharing the investment risk with farmers (Table 3).

Seven participants mentioned expectations for lead firms to help farmers upgrade their production. This was associated with improvements in productivity and product quality because rice production in Vietnam ‘was far behind in industrialization’ compared to other peer countries in the region (AE02). Current farming practice needs to be transformed and guided by ‘influential and professional organizations’ (e.g. lead firms) to ensure that the quality of agricultural products is at the required standard to penetrate strict markets (AE01). Participants also believed that, through leading and investing in production upgrades through PA adoption, lead firms could create a ‘win-win situation’ (AE01) for themselves and farmers. Farmers would find it easier to adopt the technology with ‘financial support and knowledge transfer’ (FO20) from lead firms, whereas lead firms could benefit from ‘enhanced product quality with controlled chemical residues’ (LAO01, FO14), thanks to the application of PA technology. Consequently, the linkage between farmers and lead firms is crucial in determining PA adoption for upgrades in rice production.

Some participants also emphasized the pioneering role of lead firms in pushing future PA adoption. Pioneering means that lead firms should initiate and take the lead in PA adoption for farmers to follow (Ramirez et al., 2018). We found that farmers tended to trust lead firms more than the government. They perceived that lead firms were more ‘serious in doing real business to get profit’ (AEO1, FO24), while the government only ‘propagated’ (FO15, FO13) the technological trend without any effective support.

This leads to the next significant role of lead firms in sharing the risk of technology investment with farmers. Farmers revealed that they would step back from investing in such an expensive and sophisticated technology if the associated ‘risks were not shared and embraced by a reliable organization’ (FO22). Some possible risks perceived by farmers included technological breakdown (FO03) and dissatisfied outcomes (FO16). With the good company of lead firms, farmers would be more confident to adopt, as they believed that their risk exposure could be reduced. Furthermore, ‘product offtake contracts’ (AE04) were said to be an effective incentive that lead firms should consider encouraging PA adoption among farmers. If a clear agreement exists between lead firms and farmers on the quantity and price of agricultural products that lead firms will buy at, farmers will feel safer and more confident in following the guidance of lead firms to adopt PA technology.

## Discussion

The adoption and implementation of high-tech agriculture in Vietnam have faced challenges, leading to lagging some other countries, despite the nation’s agricultural background. The predominance of traditional smallholder farmers has contributed to limited awareness and understanding of high-tech agriculture practices (Tran & Pham, 2020), and the concept of high-tech agriculture remains unfamiliar to many farmers. Our interview participants demonstrated a certain need for PA and other high-tech agricultural practices, but PA has not been widely diffused among smallholder rice farmers.

This research has overviewed the factors influencing the behavioural intention of Vietnamese smallholder rice farmers to adopt PA technology via a qualitative assessment of the opinions and perspectives of farmers and other stakeholders in the PA adoption process (e.g. technology developers, agricultural experts, and local authority representatives). This section reflects the significance of our findings in relation to the findings of previous studies to highlight the contributions to PA adoption literature and practices and points out limitations of this study and implications for future studies. The analysis has pointed to the significance of the two newly emerging themes (cooperatives’ performance and lead firm support) in addition to themes extracted from the UTAUT.

First, our findings of performance expectancy, also known as relative advantages or perceived usefulness, are in agreement with other studies in which performance expectancy is confirmed to have a positive influence on farmers’ intention to adopt PA (Adrian et al., 2005; Li et al., 2020; Walton et al., 2008; Zheng et al., 2019). Besides the expected advantages of PA technology (reducing production costs, increasing yield, efficient input management, and protecting the environment), we found that farmers looked for a better sense of control over their fields using PA technology, as well as tending to focus on potential benefits rather than the investment cost when making an adoption decision.

Second, in regard to effort expectancy, complexity and difficulty in manipulating machines and data are key constraints in adopting agricultural technologies (Kante et al., 2019; Pivoto et al., 2019). This requires a concise and simple process of technology usage for effective adoption. However, our findings revealed that, despite the technological complexities, Vietnamese rice farmers had a strong will to learn and embrace new technologies if training and consultation were available.

Third, expanding from some studies that stress the essence of government support in providing facilitating conditions to barriers to technology adoption (Zhou et al., 2010), or providing access to government financial support and consulting services as proxies for facilitating conditions (Li et al., 2020), our study attests that, despite innovation-supporting policy packages initiated by the government, in reality some farmers still had difficulties accessing them. This implies a need for policymakers to consider enhancing the exposure of supporting policies to all farmers to provide for effective practical application of PA technologies. Easier access to finance, which is facilitated by the government, was shown as important in determining the adoption of and transition to clean technologies by Yuan et al. (2022).

The extended UTAUT model, recommended by Venkatesh et al. (2016), further validates the importance of higher-level contextual factors, such as government support and policies, in influencing technology adoption decisions. In the case of Vietnam, these policy-related factors serve as a critical foundation for creating a conducive environment that fosters innovation and technological advancement across industries. Since 2009, policy packages offering subsidized credit for fertilizers, pesticides, and agricultural assets have been introduced (OECD, 2020a). Additionally, farming households benefit from agricultural land use tax reduction and exemption. Resolution No. 53/2019/NQ-CP aims to support rural development and agricultural restructuring for competitiveness and sustainability; it directs authorities to facilitate credit access and agricultural insurance programs for households in the sector (Vietnam Government Portal, 2019).

Fourth, our findings align with the related research on social influences, which argues that the way communities, peer groups, and other social influences encourage farmers’ use of PA technology affects their behavioural intention to adopt (Ajzen, 1991; Li et al., 2020). We further clarified that the peer farmers’ opinions exerted a stronger influence on the decision of Vietnamese rice farmers to adopt PA compared to advertisements by technology vendors and propaganda by the government. Technology vendors should be aware of and shift their focus to communication and information exchange between peer farmers for better promotion.

Observability and trialability are recognized as being essential in facilitating farmers’ initial exposure to the technology (Shang et al., 2020). Aubert et al. (2012) argue that trialability and adoption of PA technologies are negatively related, so that farmers may have found that the complexity of the system was beyond their prior expectations. This study confirmed that observability and trialability were helpful in reducing ambiguity and realizing the benefits of using the technology. This implies a strong effect of trialability and observability on effort expectancy and performance expectancy. In other words, if farmers had sufficient experience with the technology through trying and observing, their perception of the performance expectancy and effort expectancy would be changed accordingly.

Together with new findings on the themes of the core UTAUT model, this study has generated new themes from the qualitative data collected that critically represent the research’s theoretical contribution. These are the performance of agricultural cooperatives as innovation intermediaries and the role of lead firms. The concept of innovation intermediary has been widely discussed in innovation studies which highlight the role of innovation intermediaries in generating value from their involvement in collaborative innovation, thereby driving the progress of technology adoption (Bergek, 2020; De Silva et al., 2018; Kivimaa et al., 2020). Research in the field of PA adoption has paid less attention to examining the effect of agricultural cooperatives as innovation intermediaries. Although some studies investigate the impact of cooperative membership (e.g. whether farmers are members of cooperatives or not) on PA adoption (Barnes et al., 2019; Lampach et al., 2021; Ruzzante & Bilton, 2018; Takácsné et al., 2018), they do not provide insights into farmers’ perceptions of the effectiveness of cooperatives in supporting technology adoption, which explain their decision to join cooperatives and follow cooperatives’ guidance to adopt new technologies (Cafer & Rikoon, 2018).

To provide a deeper understanding of cooperatives’ influences on PA adoption, this study measured the performance of cooperatives as innovation intermediaries by their perceived roles in being network brokers, guiding farmers, and leading farm consolidation. As cooperatives were perceived by our participants as network brokers to link farmers with the government and lead firms, the performance of cooperatives could moderate the effect of government support and lead firm support on farmers’ behavioural intention to adopt PA technology. This opens a potential direction for further research to test the moderating effect of cooperatives’ performance as innovation intermediaries.

Another new theme emerged is that lead firms were identified by this research to pioneer PA adoption to upgrade the current farming practice and share the technology adoption risks with farmers. In the literature on value chains, lead firms often provide essential support to farmers as part of their commercial relationship with them (Ramirez et al., 2018). That support may include knowledge transfer and technical assistance (De Marchi et al., 2018). Lead firms become engaged in aggregating the production process, adding value to the products to win domestic and international markets (Ponte, 2019). Although lead firms have been often found as key innovators and respected innovation leaders in their industries (Sinkovics et al., 2021), their impacts on farmers’ behavioural intention to adopt PA technology have not been investigated. Therefore, further research on the influences of lead firms on facilitating technology adoption among farmers is needed.

Regarding the willingness to adopt PA in the near future, farmers in the South exhibited a higher intention compared to those in the North and Middle regions. An explanation for this disparity emerged from the interviews, which highlighted that farmers in the South tend to own larger farms compared to their peers in other regions. This finding aligns with recent studies on agricultural land distribution to households (Ho et al., 2022; Kiet et al., 2021). As a result, farmers with larger land holdings in the South perceive themselves to possess better resources and sufficient scale to adopt PA technology effectively.

### Theoretical Contributions

From the theoretical perspective, this study provides an empirical investigation to enrich the understanding of factors that affect the behavioural intention towards adopting PA technology among smallholder rice farmers in developing countries, which has been ignored in previous PA adoption studies. This study also opens new research directions for the PA adoption literature by primarily confirming the effect of agricultural cooperatives’ performance as innovation intermediaries and the support of lead firms on the behavioural intention of Vietnamese rice farmers to adopt PA technology.

### Practical Contributions

From a practical perspective, this study contributes to the development and diffusion of PA technologies as an emerging innovation in the agricultural practice in developing countries. The findings here will not only broaden the research agenda for later PA adoption studies but also provide a holistic way for entrepreneurial technology developers and policymakers to look at the factors that foster or hinder PA adoption. By helping enhance the understanding of PA adoption in Vietnam, this study implicitly contributes to the development of the country’s agricultural practices. With a wider adoption of PA technologies, Vietnamese farmers will have better information about their farms, which then helps them proactively optimize the inputs and reduce negative environmental impacts from farming activities. This is crucial to initiatives related to rural development towards sustainable farming goals (Rust et al., 2021).

## Conclusion

Understanding the factors affecting farmers’ behavioural intention to adopt PA technology is an essential precondition for the success of the future adoption process. In this study, we have discussed performance expectancy, effort expectancy, government support, and social influences as outstanding themes guided by the UTAUT model. The technology’s observability and trialability were found to affect farmers’ perceived performance expectancy and effort expectancy. Emerging from the interviews, the performance of agricultural cooperatives and the support of lead firms are two added themes that play a crucial role in facilitating the adoption of PA technology among Vietnamese smallholder rice farmers.

This study provides valuable insights into the factors influencing smallholder rice farmers’ behavioural intention to adopt PA technology. However, there are limitations that need to be addressed in future research. Firstly, the cross-sectional design of this study may limit its ability to capture changes in farmers’ intentions over time, especially in the context of rapid technological change and innovation. A longitudinal study would provide a more accurate understanding of how farmers’ intention to adopt PA technology changes over time.

Secondly, the sample of this study was limited to Vietnamese smallholder rice farmers, which means that the findings need to be more generalizable to large farm owners and other crop growers in Vietnam. Future studies should include a larger sample size that is more representative of the diverse range of farming practices and crop types. Factors such as farm size and crop type could also be considered as potential moderators that affect farmers’ behavioural intention to adopt PA technology.

Future research could expand on this study by testing the UTAUT model and its extensions with quantitative data analysis studies to confirm the findings and establish the robustness of the model. It could explore the influences of observability and trialability on performance expectancy and effort expectancy as extensions to the exogenous mechanism of the UTAUT. This would provide a more systematic understanding of the factors that drive farmers’ behavioural intention to adopt PA technology.

The impact of cooperatives’ performance and lead firms’ role in promoting PA technology adoption among farmers needs further attention. This could be measured and validated with appropriate quantitative techniques to establish the effectiveness of these interventions in promoting PA adoption.

Finally, it is essential to investigate the impact of PA adoption on farming outcomes such as efficiency, productivity, and sustainability. This would provide insights into the actual benefits of PA technology for farmers and the agricultural sector. Additionally, a comparative study of PA technology and conventional farming methods could also shed light on the potential advantages and disadvantages of PA technology adoption.

## Supplemental Material

Supplemental Material - Factors That Influence the Intention of Smallholder Rice Farmers to Adopt Cleaner Production Practices: An Empirical Study of Precision Agriculture Adoption Supplemental Material for Factors That Influence the Intention of Smallholder Rice Farmers to Adopt Cleaner Production Practices: An Empirical Study of Precision Agriculture Adoption by Long Le Hoang Nguyen, Duong Thuy Khuu, Alrence Halibas, and Trung Quang Nguyen in Evaluation Review
