# Supplementary material for: Factors That Influence the Intention of Smallholder Rice Farmers to Adopt Cleaner Production Practices: An Empirical Study of Precision Agriculture Adoption
Source: Eval Rev. 2023 Sep 7;48(4):692–735. doi: 10.1177/0193841X231200775 (PMC11193914; doi:10.1177/0193841X231200775)
Supplement: Supplemental Material - Factors That Influence the Intention of Smallholder Rice Farmers to Adopt Cleaner Production Practices: An Empirical Study of Precision Agriculture Adoption [file sj-pdf-1-erx-10.1177_0193841X231200775.pdf]

SUPPLEMENTARY MATERIALS

Table S1. Data structure

3rd order code (theme)	2nd order code (sub-theme)	Representative of 1st order data (in vivo)
Performance expectancy	Benefit prioritized over cost	“I think the initial investment is not the point. When a person is farming, they must buy equipment to serve their farming. Like when they bought a plow mower, they now invest in high-tech agricultural practices to save labor, precisely apply inputs, and save the environment. If the technology is effective, the farmers will invest.” (AE01).
		“If an investment promises better results than traditional farming, even if it comes at a higher price, farmers are willing to consider it.” (FO04)
		“The cost becomes negligible when we consider the efficiency of an airplane that can carry 200 liters of water and spray it over 10 hectares in just half an hour. Comparatively, using traditional methods that involve manual labor would require two days to cover the same area.” (FO12)
		“Price and quality are interlinked, as higher quality often comes with a higher cost. However, the value that a product provides determines its true worth.” (FO18)
	Enhanced efficiency	“We often fertilize according to personal experience, so sometimes the plants are also over-nutrient, which also generates fertilizer waste. If there is a technology that can know exactly how much the plant is deficient or excessive to fertilize, it will greatly increase efficiency and save cost.” (FO25).
		“Adopting precise agricultural technology is crucial to reducing costs and enhancing the quality of agricultural products. Ultimately, this leads to greater efficiency and profitability for farmers.” (AE01)
		"Advanced technology can help reduce labor costs, which is particularly important given the current trend of rising prices and a shrinking labor force in the agricultural sector." (FO11)
	Field control	“The technology I need the most is the one that helps farmers measure the amount of nutrients that a plant needs so that farmers can fertilize accurately, help rice bloom better, develop evenly, and be healthier.” (FO11)
		“The challenge is to ensure that the precision equipment used to regulate the fertilizer application is appropriate for the specific agricultural product. In the case of rice farming, the equipment must be calibrated

		to produce rice grains that meet the necessary criteria for quality and yield.” (TD03)<br>“To reduce fertilizer usage, it is essential to monitor both the living conditions of crops and the soil conditions. Additionally, proper care and management practices must be implemented to optimize the growth of the plants and minimize the need for fertilizers.” (AE02)
	Improved productivity	“As growers strive to improve their labor productivity, they inevitably increase their crop productivity. Ultimately, their primary goal is to maximize productivity in all aspects of their work.” (AE04)<br>“Farmers are willing to explore and adopt alternative methods that can boost their productivity and profitability. They are open to new ideas and innovations that can help them achieve their goals of increasing productivity and earning more revenue.” (TD02)<br>“If there are alternative approaches that can enhance their efficiency and profitability, farmers are eager to experiment with them.” (FO19)
	Improved quality	“If we want to enter big markets, our products must be clean, nice and exceptional, and to do so, we must apply and operate irrigation and cultivation technologies following the expected standards of those markets. And we must be able to monitor and control the whole process”. (AE01).<br>“By using PA, we expect to enhance the quality of agricultural products to meet the demands of global markets, particularly those with stringent requirements for microbiological standards.” (FO15)<br>“We farmers share the common goal of proactively managing our processes, improving product quality, and increasing the overall value of the agricultural output.” (FO22)
Effort expectancy	Need for training	“Technology vendors should provide training sessions on how to use it, and then farmers can communicate and instruct each other to improve their practices.” (FO17)<br>“Farmers can quickly learn to use it with just one or two instructional sessions.” (FO24)<br>“If the technology suppliers regularly send a technical team for training and knowledge transfer, farmers can feel more confident and secure in using the technology.” (TD01)
	Simple and concise process	“To ensure that farmers can easily understand and implement the knowledge, it should be presented in simple and concise processes.” (AE02)<br>“The technology should be easy to use because farmers’ knowledge of advanced technologies is not high compared to people in other industrial sectors”. (FO22).<br>“I may be hesitant to adopt technology due to concerns about the potential learning curve and operational challenges.” (FO03)

	Willingness to learn	"Although I am just a farmer with a low level of education, I am open to receiving new things. Since a young age, I have loved to discover things". (FO21)<br>"We are becoming more receptive to advanced and scientific technology products as they continue to learn and understand their benefits." (FO16)<br>"As farmers become more open-minded and receptive, it should be easier to persuade them to learn new technologies and practices". (LAO01)
Government support	Infrastructure development	"In precision agriculture, infrastructure such as roads, water supply systems, and drainage systems are essential. However, the electrical infrastructure in many production areas has not been adequately synchronized." (LAO02)<br>"Due to Vietnam's topographical characteristics, which include numerous hills and slopes, the adoption of new technology can be time-consuming." (FO24)<br>"In many fields in Vietnam, there are tombs, mounds, and hills that make it challenging to apply new technology. However, some people are hesitant to move these graves and steles, leading to tensions in some areas such as Phu Yen." (FO15)
	Plan setting	"There are still limitations in government policies and planning for fostering technology adoption in agriculture." (FO19)<br>"Vietnamese farmers are skilled in farming, and they are capable of producing good yields. However, it is crucial to have a clear master plan in place to guide the optimization their farming practices." (LAO03)<br>The state government should support farmers with clear plans for managing their agriculture. Future improvements in agriculture may not be possible without proper planning." (AE02)
	Supporting policies	"Supporting initiatives, notably financial subsidies, only go to fields owned by the state and corporations, not individual smallholder farmers like us". (FO20)<br>"We face lots of challenges in gaining access to these supporting packages." (FO16)<br>"Access to capital, policies, and technology resources is still difficult for ordinary farmers, despite the existence of relevant supporting policies." (LAO02)
Social influence	Influence of peers	"In Vietnam, farmers have a spirit of mutual affection, love, and respect for neighbors. When one person needs something, others are ready to share and help". (AE02)<br>"The testimony of other farmers who have benefited from PA technology would increase my confidence in using it. The more people talk about it, the more confident I become." (FO03)<br>"I would feel more assured to use PA technology if I hear positive feedback from other farmers who have

		benefited from it." (FO17)
	Multimedia advertisements	"Seeing advertisements about PA technology on TV and social media made me more aware of its benefits and increased my interest in trying it out." (FO04)<br>"I was skeptical about using PA technology at first, but after seeing positive reviews and testimonials from other farmers in online ads, I decided to give it a chance." (FO13)<br>"Media advertisements played a big role in convincing me to adopt PA technology". (FO18)
	Propaganda	"Farmers declined to adhere to any propaganda line. They may not understand the message. However, propaganda increases conservatism, which makes farmers deliberately not understand". (AE02).<br>"The propaganda tends to exaggerate the benefits of PA technology, which can create unrealistic expectations among farmers and lead to disappointment when they don't achieve the same results." (FO18)
Observability	Demonstration	"I think that technology needs to be demonstrated to farmers first before they can decide whether to use it or not." (FO09)<br>"We farmers are more likely to accept and adopt new technologies when we can see the positive results from other farmers who have already implemented them." (FO13)<br>"In order to convince farmers to adopt PA technology in the short term, it is necessary to demonstrate its effectiveness and showcase successful models." (FO14)
	Visibility	"In the field of agriculture in Vietnam, there is a common phenomenon where farmers tend to adopt a new technology or practice when they see others using it successfully." (TD01)<br>"We tend to follow the trend of adopting new technology or practices when we witness others successfully implementing them." (FO04)<br>"To gain farmers' trust and achieve effective technology adoption, it is crucial to choose a few points and present a sample for them to witness, as most businesses in the market erode farmers' confidence rather than building trust." (TD02)
Trialability	Experiencing model	"It is important to let us try and experience the benefits of technology before fully adopting it." (FO22)<br>"To spark farmers' interest, we need to provide demonstration models that demonstrate and prove the benefits of the technology." (AE01)<br>"Trialing is believing, and farmers need to see the benefits of using new technologies through experiencing models before they can be convinced to adopt them on a larger scale." (LAO03)
	Pre-adoption	"I am more likely to adopt PA technology if I have a positive pre-adoption evaluation of the technology's

	evaluation	potential benefits and compatibility with my existing farming practice." (FO18)<br>"A thorough pre-adoption evaluation, including assessments of the technology's advantages and disadvantages, can help me make informed decisions about whether or not to adopt PA." (FO20)
Cooperatives as innovation intermediaries	Farm consolidation	“I think small-scale is the most important hurdle. If we want to apply precision technology, we must gather small fields together into one place; it will be easier to control and apply.” (AE04).<br>"Agricultural cooperatives play a crucial role in connecting small-scale farmers, providing us with access to technology and resources that we wouldn't have on our own." (FO12)<br>"Through cooperation and collective action, agricultural cooperatives can help our small farmers adopt new technologies.” (FO15)
	Guiding farmers	"Through agricultural cooperatives, small farms like ours can learn about and apply new technologies, with the cooperative serving as an educator and guide." (FO01)<br>"Cooperatives provide a crucial link for small farms to access information, knowledge, and resources needed to adopt new technologies and improve our productivity." (FO08)<br>“Cooperatives have both advantages and disadvantages, but they are crucial for obtaining, transferring, and preserving scientific technology in a specific region, both in theory and in practice.” (LAO02)
	Network broker	“The government never provides direct capital support to farmers; they are often through to the cooperatives, agricultural extension societies or businesses.” (FO15).<br>“Cooperatives play a vital role in connecting farmers as it may not be feasible for businesses to meet with each individual farmer for discussions.” (FO12)<br>“The cooperative is supposed to be the entity that safeguards the interests of farmers and acts as their representative to seek support from businesses and the government. However, it appears that the cooperative has not performed this role satisfactorily.” (FO24)
Role of lead firms	Upgrading production	"Lead firms have a responsibility to support small farms in adopting technology for sustainable supply chains, as it benefits not only the farmers but also the overall business ecosystem." (AE03)<br>"Collaborating with small farms to adopt technology is a win-win situation for lead firms, as it can improve supply chain efficiency, reduce costs, and enhance sustainability." (LAO03)<br>"By supporting small farms in technology adoption, lead firms can play a pivotal role in transforming the agricultural sector towards a more sustainable and equitable future." (TD01)
	Pioneering	“The adoption of high technology necessitates that businesses take the lead.” (AE04)

		“If businesses take the lead in collaborating with the government to make technology accessible to farmers and create favorable conditions for them, Vietnam has the potential to effectively adopt and utilize this technology.” (LAO01)<br><br>“It is primarily the responsibility of businesses and cooperatives to actively reach out to farmers and encourage them to adopt new practices and technologies.” (FO24)
	Risk sharing	"Lead firms that share the risk of technology investment with small farms can build stronger partnerships and drive innovation for sustainable agricultural practices." (FO03)<br><br>"By sharing the risk of technology investment with small farms, lead firms can enable them to adopt advanced practices and compete in the global marketplace." (FO16)<br><br>"Collaborative technology investment between lead firms and small farms not only creates shared value but also enhances the resilience and sustainability of the entire agricultural supply chain." (AE01)
